# Leveraging genetic correlation structure to target discrete signaling mechanisms across metabolic tissues

**DOI:** 10.1101/2023.05.10.540142

**Published:** 2023-05-12

**Authors:** Mingqi Zhou, Cassandra Van, Jeffrey Molendijk, Ivan Yao-Yi Chang, Casey Johnson, Leandro M. Velez, Reichelle X. Yeo, Hosung Bae, Johnny Le, Natalie Larson, Ron Pulido, Carlos H V Nascimento-Filho, Andrea Hevener, Lauren M. Sparks, Jaime N. Justice, Erin E. Kershaw, Ivan Marazzi, Nicholas Pannunzio, Dequina Nicholas, Benjamin Parker, Cholsoon Jang, Selma Masri, Marcus Seldin

**Affiliations:** 1 Department of Biological Chemistry, UC Irvine. Irvine, CA, USA; 2 Center for Epigenetics and Metabolism, UC Irvine. Irvine, CA, USA; 3 Department of Anatomy and Physiology, University of Melbourne, Melbourne, VIC, Australia; 4 Translational Research Institute, AdventHealth, Orlando, FL, USA; 5 Department of Medicine, Division of Endocrinology, Diabetes, and Hypertension, David Geffen School of Medicine at UCLA Los Angeles, CA, USA; 6 Iris Cantor-UCLA Women’s Health Research Center, David Geffen School of Medicine at UCLA Los Angeles, CA, USA; 7 Veterans Administration Greater Los Angeles Healthcare System, Geriatric Research Education and Clinical Center (GRECC), Los Angeles, CA, USA; 8 Department of Internal Medicine, Section On Gerontology and Geriatric Medicine, Wake Forest School of Medicine, Winston-Salem, NC, USA; 9 Division of Endocrinology, Department of Medicine, University of Pittsburgh, Pittsburgh, Pennsylvania, USA; 10 Divison of Hematology/Oncology, Department of Medicine, University of California Irvine, Irvine, CA USA; 11 Department of Molecular Biology and Biochemistry, School of Biological Sciences, University of California Irvine, Irvine CA, USA

**Keywords:** Endocrinology, Inter-tissue communication, Organ cross-talk, Systems genetics, Genetic variation, Web resource

## Abstract

Inter-organ communication is a vital process to maintain physiologic homeostasis, and its dysregulation contributes to many human diseases. Beginning with the discovery of insulin over a century ago, characterization of molecules responsible for signal between tissues has required careful and elegant experimentation where these observations have been integral to deciphering physiology and disease. Given that circulating bioactive factors are stable in serum, occur naturally, and are easily assayed from blood, they present obvious focal molecules for therapeutic intervention and biomarker development. For example, physiologic dissection of the actions of soluble proteins such as proprotein convertase subtilisin/kexin type 9 (*PCSK9*) and glucagon-like peptide 1 (*GLP1*) have yielded among the most promising therapeutics to treat cardiovascular disease and obesity, respectively^1–4^. A major obstacle in the characterization of such soluble factors is that defining their tissues and pathways of action requires extensive experimental testing in cells and animal models. Recently, studies have shown that secreted proteins mediating inter-tissue signaling could be identified by “brute-force” surveys of all genes within RNA-sequencing measures across tissues within a population^5–9^. Expanding on this intuition, we reasoned that parallel strategies could be leveraged to understand how individual genes mediate signaling across metabolic tissues through correlative analysis of genetic variation. Thus, genetics could aid in understanding cross-organ signaling by adopting a gene-centric approach. Here, we surveyed gene-gene genetic correlation structure for ~6.1×10^^12^ gene pairs across 18 metabolic tissues in 310 individuals where variation of genes such as *FGF21, ADIPOQ, GCG* and *IL6* showed enrichments which recapitulate experimental observations. Further, similar analyses were applied to explore both local signaling mechanisms (liver *PCSK9*) as well as genes encoding enzymes producing metabolites (adipose *PNPLA2*), where genetic correlation structure aligned with known roles for these critical metabolic pathways. Finally, we utilized this resource to suggest new functions for metabolic coordination between organs. For example, we prioritized key proteins for putative signaling between skeletal muscle and hippocampus, and further suggest colon as a central coordinator for systemic circadian clocks. We refer to this resource as **G**enetically-**D**erived **C**orrelations **A**cross **T**issues (GD-CAT) where all tools and data are built into a web portal enabling users to perform these analyses without a single line of code (gdcat.org). This resource enables querying of any gene in any tissue to find genetic coregulation of genes, cell types, pathways and network architectures across metabolic organs.

## Results

### Genetic correlation structure recapitulates established mechanisms of metabolic signaling –

**Figure 1.**
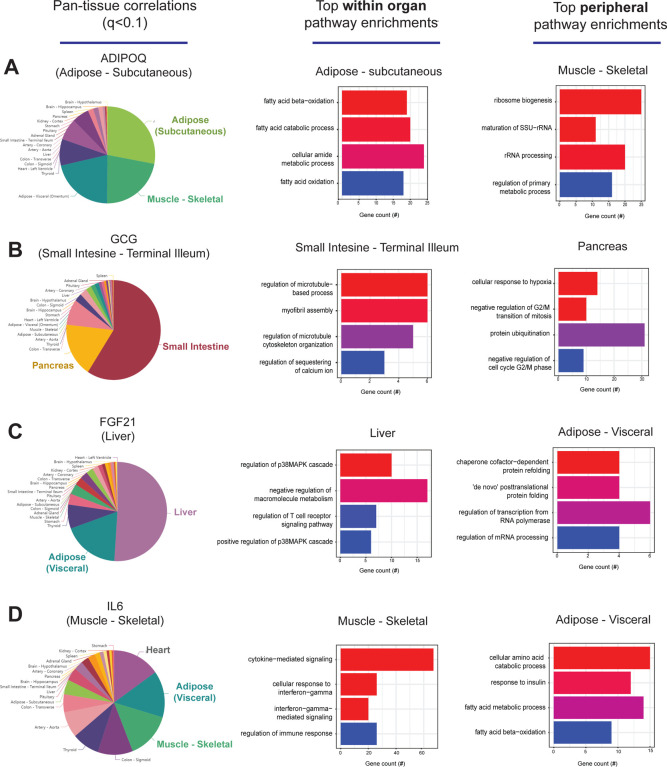
Genetic correlation structure of established endocrine proteins. A, All genes across the 18 metabolic tissues in 310 individuals were correlated with expression of *ADIPOQ* in subcutaneous adipose tissue, where a qvalue cutoff of q<0.1 showed the strongest enrichments with local subcutaneous and muscle gene expression (pie chart, left). The top 500 genes which correlated with subcutaneous *ADIPOQ* were used for an overrepresentation test across Gene Ontology Biological process annotations, where pathways related to fatty acid oxidation were observed in adipose (middle) and ribosome/metabolic processes in skeletal muscle (right). B-D, The same qvalue binning, local and top peripheral enrichments were applied to intestinal *GCG* (B), liver *FGF21* (C) and muscle *IL6* (D). For these analyses all 310 individuals (across both sexes) were used and qvalue adjustments calculated using a Benjamini-Hochberg FDR adjustment.

**Figure 2.**
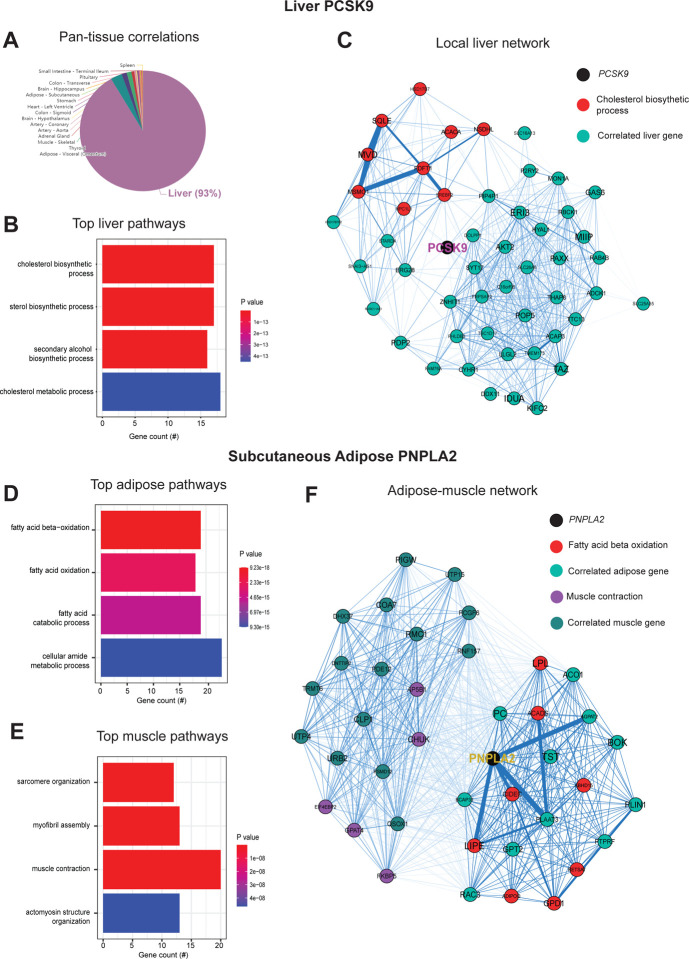
Genetic correlation structure and network architecture of liver *PCSK9* and adipose *PNPLA2*. A, distribution of pan-tissue genes correlated with liver *PCSK9* expression (q<0.1), where 93% of genes were local in liver (purple). B, Gene ontology (BP) overrepresentation test for the top 500 hepatic genes correlated with *PCSK9* expression in liver. C, Undirected network constructed from liver genes (aqua) correlated with *PCSK9*, where those annotated for “cholesterol biosynthetic process” are colored in red. D-E, over-representation tests corresponding to the top-correlated genes with adipose (subcutaneous) *PNPLA2* expression residing locally (D) or peripherally in skeletal muscle (E). F, Undirected network constructed from from the strongest correlated subcutaneous adipose tissue (light aqua) and muscle genes (dark blue) with PNPLA2 (black), where genes corresponding to GO terms annotated as “fatty acid beta oxidation” or “Muscle contraction” are colored purple or red, respectively. For these analyses all 310 individuals (across both sexes) were used and qvalue adjustments calculated using a Benjamini-Hochberg FDR adjustment. Network graphs generated based in Biweight midcorrelation coefficients, where edges are colored blue for positive correlations or red for negative correlations.

Previous studies have established that “brute force” analyses of genetic correlation structure across tissues can identify new mechanisms of organ cross-talk. These were accomplished by surveying the global regression structure using all genes, whereby skewed upper-limits of significance distributions were sufficient to prioritize proteins which elicit signaling^5–9^. Following this intuition, we hypothesized that a paralleled but alternative approach to genetic correlation structure could be exploited to understand the functional consequences of specific genes. To test this hypothesis, we initially examined pan-tissue genetic correlation structures for several well-established mechanisms of tissue crosstalk via secreted proteins which contribute significantly to metabolic homeostasis. We utilized the most comprehensive pan-tissue dataset in humans (GTEx)^10^, which was filtered for individuals where most metabolic tissues were sequenced^9^. Collectively, this dataset contains 310 individuals, consisting of 210 male and 100 female (self-reported) subjects between the ages of 20–79. Gene correlation structure showed strong overlap with known physiologic roles of given endocrine proteins. For example, variation with subcutaneous adipose expression of *ADIPOQ* was enriched with genes in several metabolic tissues where it has been known to act (Fig 1A, left). In particular, subcutaneous adipose *ADIPOQ* expression correlated with fatty acid oxidative process locally in adipose (Fig 1A, middle) and was enriched with ribosomal biogenesis in skeletal muscle (Fig 1C, right). These genetic observations align with the established physiologic roles of the protein in that fat secreted adiponectin when oxidation is stimulated^11,12^ and muscle is a major site of action^13^. Beyond adiponectin, genetic correlation structure additionally recapitulated broad signaling mechanisms for other relevant endocrine proteins. For example, intestinal *GCG* (encoding GLP1), liver *FGF21* and skeletal muscle *IL6* showed binning patterns and pathway enrichments related to their known functions in pancreas^1,14^, adipose tissue^15,16^ and other metabolic organs^17^, respectively. Next, we wanted to ask whether our approach of analyzing genetic correlation structure across tissue for endocrine proteins was also sufficient to define local signaling mechanisms or actions of enzymes producing metabolites that signal in circulation. Dissimilar to the cross-tissue distributions of significance in Fig 1, the same analysis of liver *PCSK9* highlighted exclusively local genes which were genetically coregulated (Fig 2A), in particular those involved in cholesterol metabolism/homeostasis (Fig 2B). Consistent with the established role for PCSK9 as a primary degradation mechanism of LDLR^4,18^, network model construction of genetically coregulated genes highlighted the gene as a central node linking cholesterol biosynthetic pathways with those involved in other metabolic pathways such as insulin signaling (Fig 2C). Given that organ signaling via metabolites comprises many critical processes among multicellular organisms, our next goal was to apply this gene-centric analyses to established mechanisms of metabolite signaling. The gene *PNPLA2* encodes adipose triglyceride lipase (ATGL) which localizes to lipid droplets and breaks down triglycerides for oxidation or mobilization as free fatty acids for peripheral tissues^19^. Variation in expression of PNPLA2 showed highly significant local enrichments with beta oxidation pathways in adipose tissue (Fig 2D). Muscle pathways enriched for the gene were represented by sarcomere organization and muscle contraction (Fig 2F). Construction of an undirected network from genetic data placed the gene as a central node between the two tissues, linking regulators of adipose oxidation (Fig 2F, red) to muscle contractile process (Fig 2F, purple) where additional strongly co-correlated genes were implicated as additional candidates (Fig 2F). In sum, these analyses demonstrate that simple surveys of genetic correlation structure using a gene-centric approach recapitulates established aspects of metabolic signaling both within and across organs.

### Focused analyses of genetic correlation structure identifies pan-tissue links between circadian clocks.

**Figure 3.**
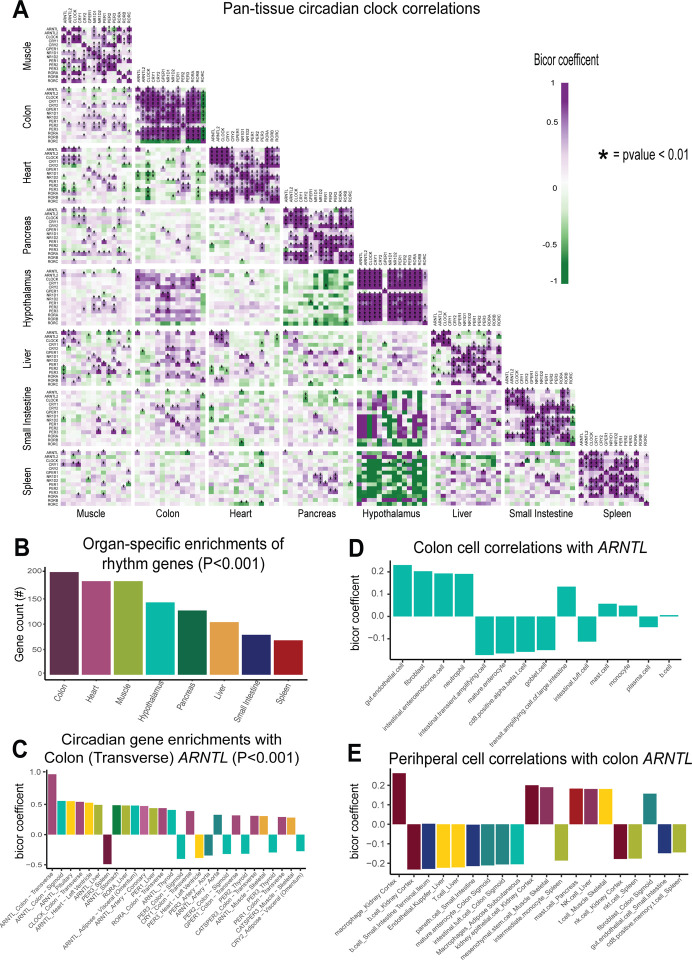
Pan-tissue circadian clock concordances highlights centrality of colon *ARNTL*. A Heatmap showing Biweight midcorrelation coefficients between key circadian rhythm genes across metabolic tissues. Positive correlations are shown in purple negative correlations in green, where a * indicates significance (regression Pvalue<0.01). B, Frequency (y-axis) of circadian rhythm genes per tissue (x-axis), where regression P value was less than 0.001 across all other rhythm genes in other tissues. C, The top-ranked rhythm genes (x-axis) based on biweight midcorrelation (y-axis) with expression of *ARNTL* in colon. D-E, The top-ranked cell types (x-axis) correlated with colon ARNTL expression based on Biweight midcorrelation coefficient (y-axis), either locally within colon (D), or across peripheral tissue deconvoluted cell abundances (E). For these analyses all 310 individuals (across both sexes) were used and regression pvalues calculated from the bicor coefficient using WGCNA^41^.

Following confirmation that a gene-centric approach to analysis of genetic correlation structure was sufficient to recapitulate discrete physiologic mechanisms, our next goal was to leverage this approach to understand new modes of coordination between metabolic organs. Shifts in biological rhythms have been associated with nearly every aspect of maintenance of metabolic homeostasis and associated diseases, where in many experimental settings have been demonstrated to play causal roles^20,21^. Oscillations in circadian rhythms have recently been shown to be tightly-linked across tissue^22^ where communication between clocks is perturbed in states such as high-fat feeding^23,24^. To evaluate genetic concordance of tissue-specific clocks, we surveyed co-correlation structure between organ clock genes in the same 310 individuals (Fig 3A). While defining mechanisms of circadian rhythms require careful control of temporal collection, it is worth noting that recent analysis of GTEx unveiled modes of sex-specific temporal expression^25^. In our analysis focused simply on genetic variation, clock genes appeared more strongly correlated within, rather than between tissues. While most rhythmic genes appeared positively correlated (purple) across tissues, several areas of strong negative correlations appeared such as between hypothalamus and spleen or pancreas (Fig 3A). To define which tissue clocks showed the strongest level of average concordance of rhythms systemically, clock gene correlations were summarized by counting the number of significant correlations (regression Pvalue <0.001) across all tissues. This analysis showed that colon and heart clock genes appeared top-ranked and thus, most central in their correlation with rhythm genes in other peripheral tissues (Fig 3B). Consistent with this observation, the core clock transcriptional regulator, BMAL1 (*ARNTL*) was significantly correlated with *ARNTL* across nearly every metabolic tissue (Fig 3C). In colon, *ARNTL* has been demonstrated to play key roles in systemic metabolism^26,27^, where disruption leads to severe consequences such as cancer^28,28^. For example, genetic ablation of *ARNTL* in mice exacerbates colon cancer development through loss-of-heterozygosity of APC^29^. To further refine what cell types might be involved, bulk sequencing was subjected to single-cell deconvolution (methods) where cell types were correlated with gene expression in the 310 individuals. Within colon, *ARNTL* was most strongly enriched with endothelial cells, enteroendocrine cells and fibroblasts (Fig 3D); while outside of colon the gene showed enrichments with kidney, small intestine and liver (Fig 3E). These analyses suggest colon as an important clock-controlled tissue, where variation in *ARNTL* expression is strongly correlated with peripheral clocks and relevant metabolic cell types such as gut enteroendocrine cells and kidney macrophages. Given the nature of enteroendocrine cells in regulating hormones such as GLP1, these interactions with biological rhythms merit further investigation.

### Genetic correlation analysis prioritizes proteins involved in muscle-brain signaling.

**Figure 4.**
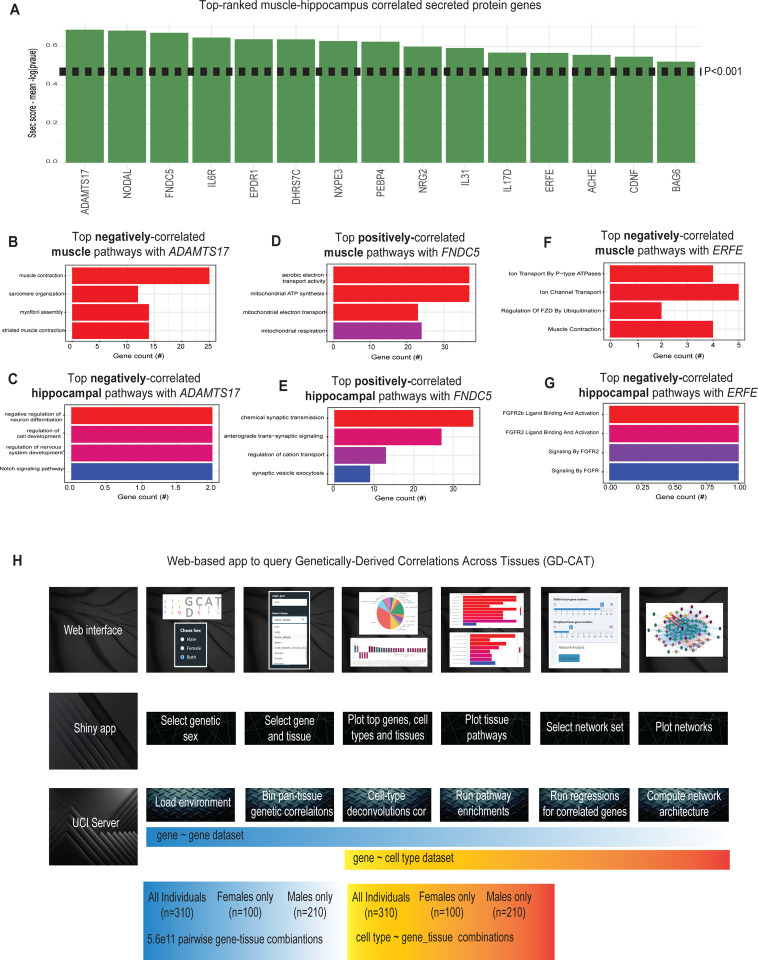
Putative muscle-hippocampal brain signals and web tool overview. A, Genes encoding secreted proteins (x-axis) plotted against the average -log(regression-pvalue) with all transcripts in hippocampus (y-axis), termed Ssec score^5^. B-G, For select genes shown in A, the pathway enrichments from overrepresentation tests based on the top 500 genes within skeletal muscle (B, D, F) or Hippocampus (C, E, G) are shown for ADAMTS17 (B-C), FNDC5 (D-E) or ERFE (F-G). H, Schematic for design of web portal to query all gene-gene and gene-cell genetic correlations and run targeted analyses.

Communication between muscle and brain has attracted significant recent attention as a potential mechanistic link between the beneficial effects of exercise and improved cognitive outcomes^30,31^. For example, muscle-expressed proteins BDNF^32,33^ and FNDC5/Irisin^34,35^ have been shown to enhance learning and memory in mice models. To uncover additional putative endocrine factors involved in muscle-brain signaling, we surveyed genetic correlation structure between all muscle genes encoding secreted proteins^5,7–9^ and expression variation in the hippocampus (Fig 4A). Among the top-ranked genes, previously-established myokines were observed such as *FNDC5* and *IL6*. Application of gene-centric analyses highlighted select muscle-hippocampal pathways which were enriched with signaling mechanisms. For example, expression of muscle ADAMTS17 was negatively correlated with local contraction-associated pathways (Fig 4B) and neuron development pathways in hippocampus (Fig 4C). Dissimilar to ADAMTS17 and consistent with its established roles in muscle-brain signaling, muscle-expressed FNDC5 was positively associated with respiratory pathways in muscle (Fig 4D) and chemical synaptic signaling in brain (Fig 4E). In addition, myonectin/ERFE showed a link between negatively-correlated ion flux (Fig 4F) in muscle and FGF-mediated signaling in brain (Fig 4G). While ADAMTS17 and myonectin/ERFE have been shown to regulate skeletonegesis^36^ and liver metabolism/iron homeostasis^37–40^, the suggested links to brain, hippocampus and neurons derived from genetic correlations presents potentially novel signaling mechanisms.

### Construction of a web tool to survey genetically-derived correlations across tissues (GD-CAT).

These analyses demonstrate that systematic surveys of genetic correlation structure across metabolic tissues are sufficient to recapitulate several known and potentially novel modes of inter-tissue signaling. Our next goal was to establish a user-friendly interface where all of these analyses and gene-centric queries could be performed without writing a single line of code. To accomplish this, we assembled a complete analysis pipeline (Fig 4H) as a shiny-app and docker image hosted in a freely-available web address (gdcat.org). Here, users can readily-search genetic correlation structure from filtered GTEx data across tissues. Initially, users select a given genetic sex (or combines both) which loads the specified environment, where subsequent downstream analyses are implemented. Next, a specific gene is selected from one of the 18 tissues listed, which is then correlated cross all other gene-tissue combinations (748,280 total) using Biweight midcorrelation^41^. Next, Benjamini-Hochberg FDR adjustments are applied to the distribution of regression p-values where pie charts provide the tissue-specific occurrences of correlated genes at 3 thresholds (q<0.1, q<0.01, q<0.001). From these charts, users are able to select a given tissue, where overrepresentation tests using enrichR^42^ are applied to the strongest positively and negatively correlated genes. Our previous studies suggested that usage of the top 500 genes demonstrated a relatively informative pathway enrichment profile^5^, so the number is capped at 500 for a given qvalue threshold. In addition to general queries of gene ~ gene correlation structure, we included the top cell-type abundance correlations with each gene as well. To compute cell abundance estimates from the same individuals, we used single-nucleus RNA-seq available from GTEx^43^ and applied cellular deconvolution methods to the bulk RNA-seq^44^. Comparison of deconvolution methods^44^ showed that DeconRNASeq^45^ captured the most cell types within visceral adipose, subcutaneous adipose, aortic artery, coronary artery, transverse colon, sigmoid colon, the heart left ventricle, the kidney cortex, liver, lung, skeletal muscle, spleen, and small intestine.(Supplemental Figures 1–3) and therefore was applied to all tissues where sn-RNA-seq was available. The inferred abundances of cell types from each individual are correlated across user-defined genes, with the bicor coefficient plotted for each cell type. We note that sn-RNA-seq within the brain, stomach and thyroid are not available within the human cell atlas and therefore, not present in these analyses.

## Discussion

### Limitations and Conclusions –

Several key limitations should be considered when exploring GD-CAT for mechanisms of inter-tissue signaling. Primarily, the fact that correlation-based analyses could reflect both causal or reactive patterns of variation. While several statistical methods such as mediation^46,47^ and mendelian randomization^48,49^ exist to further refine causal inferences, likely the only definitive method to distinguish is in carefully-designed experimentation. Further, pan-tissue correlations tend to be dominated by local regressions where a given gene is expressed. This is due to the fact that within-tissue correlations could capture both the regulatory and putative consequences of gene regulation, and distinguishing between the two presents a significant challenge. In addition, the analyses presented are derived from genetic differences of gene expression. Expression of a gene and its corresponding protein can show substantial discordances depending on the dataset used. We note that for genes encoding proteins where actions from acute secretion grossly outweigh patterns of gene expression, such as insulin, caution should be taken when interpreting results. As the depth and availability of tissue-specific proteomic levels across diverse individuals continues to increase, an exciting opportunity is presented to explore the applicability of these analyses and identify areas when gene expression is not a sufficient measure.

The queries provided in GD-CAT use fairly simple linear models to infer organ-organ signaling; however, more sophisticated methods can also be applied in an informative fashion. For example, Koplev et al generated co-expression modules from 9 tissues in the STARNET dataset, where construction of a massive Bayesian network uncovered interactions between genetically correlated modules^6^. Further, development of MultiCens, a tool to uncover communication between genes and tissues has helped to determine central processes which exist in multi-layered data relevant for Alzheimer’s disease^50^. In addition, Jadhav and colleagues adopted a machine learning approach to mine published literature for relationships between hormones and genes^51^. Further, association mapping of plasma proteomics data has been extensively applied and intersection with genome-wide association disease loci has offered intriguing potential disease mechanisms^52,53^. Another common application to single-cell sequencing data is to search for overrepresentation of known ligan-receptor pairs between cell types^54^. These and additional applications to explore tissue communication/coordination present unique strengths and caveats, depending on the specific usage desired.

In sum we demonstrate that adopting a gene-centric approach to surveying genetic correlation structure can inform mechanism of coordination between metabolic tissues. Initially, we queried several well-establish and key mediators of physiologic homeostasis, such as *FGF21, GCG* and *PCSK9*. These approaches are further suggested to be applicable to mechanisms of metabolite signaling, as evident by pan-tissue investigation of adipose *PNPLA2*. Beyond recapitulation of established mechanisms of tissue communication, similar approaches can be applied to uncover genetic patterns of potentially new signaling. For example, our analyses suggest colon as a central node of circadian rhythm genes and prioritized putative signaling between muscle and brain. To facilitate widespread access and use of this gene-centric analysis of genetic correlation, a full suite of analyses such as those performed here can be performed from a lab-hosted server (gdcat.org) or in isolation from a shiny app or docker image.

## Material and methods

### Availability of web tool and analyses:

All analyses, datasets and scripts used to generate the associated web tool (GD-CAT) can be accessed via: https://github.com/mingqizh/GD-CAT or within the associated docker image. In addition, access to the GD-CAT web tool is also available through the web portal gdcat.org. This portal was created to provide a user-friendly interface for accessing and using the GD-CAT tool without the need to download or install any software or packages. Users can simply visit the website, process data and start using the tool. Corresponding tutorial and the other resources were made available to facilitate the utilization of the web tool on GitHub. The interface and server of the web were built and linked based on the shiny package using R (v. 4.2.0). Shiny package provides a powerful tool for building interactive web applications using R, allowing for fast and flexible development of custom applications with minimal coding required.

### Data sources and availability –

All data used in this study can be immediately accessed via web tool or docker to facilitate analysis. Metabolic tissue data was accessed through GTEx V8 downloads portal on August 18, 2021 and previously described^9,10^. These raw data can also be readily accessed from the associated R-based walkthrough: https://github.com/Leandromvelez/myokine-signaling. Briefly, these data were filtered to retain genes which were detected across tissues where individuals were required to show counts > 0 in 1.2e6 gene-tissue combinations across all data. Given that our goal was to look across tissues at enrichments, this was done to limit spurious influence of genes only expressed in specific tissues in specific individuals.

### Selection of secreted proteins and circadian rhythm genes –

To determine which genes encode proteins known to be secreted as myokines (Fig 4), gene lists were accessed from the Universal Protein Resource which has compiled literature annotations terms for secretion^55^. Specifically, the query terms to access these lists were: locations:(location:”Secreted [SL-0243]” type:component) AND organism:”Homo sapiens (Human) [9606]” where 3666 total entries were found. Genes encoding relevant circadian rhythm regulators (Fig 3) were used from manually-curated lists and previously described^29^.

### Regression analyses across tissues –

Regression coefficients and corresponding p-values within and across tissues were generated using WGCNA bicorandpvalue() function^41^. Associated qvalue adjustments were applied using the Benjamini-Hochberg FDR from the R package “stats”.

### Pathway enrichment analyses –

Pathway enrichments were generated using overrepresentation tests among Gene Ontology (Biological Process) and Reactome databases using the R package enrichR. For this analysis, upper limits of 500 genes were set to perform enrichments where if a lesser number occurred at indicated qvalue threshold, the maximum number of genes was used.

### Deconvolution of bulk tissue seq data.

All scripts and deconvolution data produced is available at: https://github.com/cvan859/deconvolution. Briefly, sn-RNA-seq data was accessed from the Human cell atlas^43^ for matching organ datasets with metabolic tissues. From these data, 4 deconvolution methods were applied using ADAPTS^44^ where DeconRNA-Seq^45^ was selected for its ability to capture the abundances of the most cell types across tissues such as liver heart and skeletal muscle (Supplemental Fig 1–3). The full combined matrix was assembled for DeconRNA-Seq results across individuals in GTEx where correlations between cell types and genes was performed also using WGCNA^41^.
