# Supplementary material for: Leveraging inter-individual transcriptional correlation structure to infer discrete signaling mechanisms across metabolic tissues
Source: bioRxiv. 2023 Oct 4:2023.05.10.540142. Originally published 2023 May 12. Preprint. [Version 2] doi: 10.1101/2023.05.10.540142 (PMC10197628; doi:10.1101/2023.05.10.540142)
Supplement: Supplement 1 [file NIHPP2023.05.10.540142v2-supplement-1.pdf]

bioRxiv preprint doi: https://doi.org/10.1101/2023.05.10.540142; this version posted October 4, 2023. The copyright holder for this preprint (which was not certified by peer review) is the author/funder, who has granted bioRxiv a license to display the preprint in perpetuity. It is made available under aCC-BY 4.0 International license.

Supplemental Figure 1: Performance across 4 methods of cell-type deconvolution where relative proportions of cells (y-axis) are shown for all cell types annotated in single-cell reference (x-axis) in Liver.

Supplemental Figure 2: Performance across 4 methods of cell-type deconvolution where relative proportions of cells (y-axis) are shown for all cell types annotated in single-cell reference (x-axis) in Heart.

Supplemental Figure 3: Performance across 4 methods of cell-type deconvolution where relative proportions of cells (y-axis) are shown for all cell types annotated in single-cell reference (x-axis) in Skeletal Muscle.

Supplemental Figure 4: Pancreatic *INS* expression correlations across tissues in GTEx were binned according to $q<0.1$ (top) and corresponding pancreatic GSEA network graph is shown (bottom)
